# Long-term high loading intensity of aerobic exercise improves skeletal muscle performance *via* the gut microbiota-testosterone axis

**DOI:** 10.3389/fmicb.2022.1049469

**Published:** 2022-12-21

**Authors:** Lidong Zhang, Hedong Lang, Li Ran, Guoliang Tian, Hui Shen, Jundong Zhu, Qianyong Zhang, Long Yi, Mantian Mi

**Affiliations:** Chongqing Key Laboratory of Nutrition and Food Safety, Research Center for Nutrition and Food Safety, Chongqing Medical Nutrition Research Center, Institute of Military Preventive Medicine, Army Medical University (Third Military Medical University), Chongqing, China

**Keywords:** exercise, gut microbiota, testosterone, skeletal muscle, grip strength

## Abstract

Exercise is reported to play a crucial role in skeletal muscle performance. However, the underlying mechanism is still unknown. Thus, we investigated the effect of high-intensity aerobic exercise on skeletal muscle performance. In this study, the male C57BL/6J mice were accepted by high-intensity aerobic exercise for 8 weeks to establish an exercise model. It was observed that high-intensity aerobic exercise markedly affected the expression of genes in skeletal muscle. Moreover, high-intensity aerobic exercise significantly improved skeletal muscle grip strength and serum testosterone levels. HE staining showed that the cross-sectional area (CSA) of the skeletal muscle was successfully increased after 8 weeks of high-intensity aerobic exercise. Additionally, we found that high-intensity aerobic exercise changed gut microbiota structure by altering the abundance of *Akkermansia*, *Allobaculum*, and *Lactobacillus*, which might be related to testosterone production. However, the beneficial effects disappeared after the elimination of the gut microbiota and recovered after fecal microbiota transplantation (FMT) experiments for 1 week. These results indicated that the beneficial effects of high-intensity aerobic exercise on skeletal muscle were partly dependent on the gut microbiota. Our results suggested that long-term high loading intensity of aerobic exercise could improve skeletal muscle performance, which was probably due to the gut microbiota-testosterone axis.

## Introduction

Skeletal muscle performance is crucial for athletes. Specifically, evidence suggests that increasing the muscle’s CSA could greatly impact the muscle’s ability (Suchomel et al., 2018). And exercise training could significantly induce skeletal muscle hypertrophy (Bahreinipour et al., 2018; Torma et al., 2021), which are well-known treatments for athletes to improve skeletal muscle performance. Recently we have shown that exercise has been linked to serum testosterone levels. Moderate-intense exercise and resistance training (Vingren et al., 2010) could improve serum testosterone levels (Sharma et al., 2013; Khajehnasiri et al., 2018). However, some studies indicated that aerobic exercise could also induce skeletal muscle hypertrophy (Harber et al., 2009, 2012; Konopka and Harber, 2014), but the underlying mechanism is still unknown.

Testosterone is an anabolic hormone regulated by the hypothalamic–pituitary-testicular axis (HPA; Bélanger et al., 2003; Anderson et al., 2018). Testosterone is produced mainly by the testis, metabolized by the liver, and reabsorbed by the intestinal tract (Sandberg and Slaunwhite, 1956; Baulieu, 1991; Li et al., 2022). Testosterone plays a crucial role in satellite cell activation, conversion of fiber, and synthesis of protein (Leproult and Van Cauter, 2011; Carson and Manolagas, 2015; Rossetti et al., 2017). Furthermore, low endogenous testosterone production cause muscle wasting, and injection of testosterone has been shown to improve muscle mass and strength (Falqueto et al., 2021; Harper et al., 2021; Alexander et al., 2022). Thus, serum testosterone levels of athletes are crucial for their skeletal muscle performance.

Gut microbiota is one of the most attractive research focuses in recent years (Heintz-Buschart and Wilmes, 2018; Whon et al., 2021). Exercise could alter the gut microbiota’s structure and affect the metabolic function of skeletal muscle (Frampton et al., 2020). Recent studies suggest that gut microbiota is related to testosterone metabolism (Liu et al., 2017; Colldén et al., 2019). The gut microbiota could convert T to dihydrotestosterone (DHT) and cause deglucuronidation of glucuronidated testosterone (T-G) (Soory, 1995; Colldén et al., 2019). Moreover, the serum testosterone levels in germ-free mice are lower (Markle et al., 2013), but the mice fed with *Lactobacillus* significantly increase serum testosterone levels compared with mice fed with a normal diet (Poutahidis et al., 2014). Lack of testosterone causes depression. The 3β-hydroxysteroid dehydrogenase expressed by gut microbes degrades testosterone which has been implicated in male depression (Li et al., 2022). What’s more, some studies have demonstrated that *Akkermansia* was positively correlated with serum testosterone levels (Zhang et al., 2021). Unfortunately, the effects of gut microbiota on testosterone metabolism are unclear.

In this study, we aimed to observe the effect of high-intensity aerobic exercise on skeletal muscle performance. Unexpectedly, we found that exercise could improve skeletal muscle performance by altering the gut microbiota’s structure (*Lactobacillus, Allobaculum,* and *Akkermansia*) and testosterone metabolism. Thus, our findings demonstrated that high-intensity aerobic exercise could improve skeletal muscle performance *via* the gut microbiota-testosterone axis. Together, we provided new insights into the role of gut microbiota in testosterone metabolism and offered new opportunities into improving skeletal muscle performance for athletes.

## Materials and methods

### Experimental design

The male C57BL/6J mice (7 weeks, 19–21 g) were purchased from the Laboratory Animal Centre of the Army Medical University (Chongqing, China). Mice were kept 4 per cage in a controlled environment (22°C–25°C, 50%–55% humidity, 12 h light/dark cycle) for 7 days before the experiment. All mice obtained the water and standard laboratory chow diet freely (Chen et al., 2019). Animal experiments included two parts (*n* = 12/group). Part 1: CON, Abx, EX, and EX + Abx. Mice in EX groups were accepted by exercise training for 8 weeks, and Abx groups were subjected to the Abx treatment for 1 week before the training. Part 2: CON-donor and EX-donor. Mice in donor groups were performed by FMT experiment for 1 week. We recorded the body weight and food intake every week.

### Tissue isolation

We sacrificed the mice 24 h after the last training. The blood taken from the eyeball was centrifugation (3,000 rpm, 15 min, 4°C) to collect the serum. The small intestine, gastrocnemius muscles (GA), cecum content, and testis were collected and stored at −80°C. All animal studies were approved by the Animal Care and Use Committee of the Army Medical University.

### Exercise training protocol

The EX groups were subjected to exercise on the motorized treadmill (Jiangsu, China). Mice were adapted to the treadmill environment for 1 week (0°, 15 m/min, 10 min; Fernando et al., 1993; Zhang et al., 2022). Then, according to Bedford’s method (Bedford et al., 1979; E et al., 2013; Kim et al., 2015), mice were randomly assigned to high-intensity aerobic exercise (10°, 20 m/min, 60 min, 5 day/week) for 8 weeks.

### Antibiotic treatment and FMT experiments

Antibiotic treatment: mice were subjected to the antibiotic cocktail (Abx) (0.5 g/L vancomycin, 1 g/L ampicillin, 1 g/L metronidazole, and 1 g/L neomycin sulfate) for 1 week as literature reported (Miyauchi et al., 2020; Hui et al., 2020a; Secombe et al., 2021; Li et al., 2022). FMT experiments: we collected the fresh fecal from mice in CON and EX groups after the last intervention. The fecal (50 mg) was dissolved in 2.5 ml PBS, shaken (2 min), and centrifuged (10,000 g, 10 min, 4°C) to collect the supernatant. The mice in donor groups were treated with the Abx treatment for 1 week, then administered with the above 200 μl supernatant for 1 week (Sun et al., 2018). And the effects of gut microbiota elimination and FMT were confirmed by stool DNA concentration analysis. We collected fecal samples at 1 day before antibiotic treatment, 1 week after antibiotic treatment, and 1 week after FMT experiment.

### Grip strength test

The muscle grip strength of the mice was measured by a grasping instrument (Ugo Basile, Italy) 24 h after the last exercise training (Huang et al., 2021). With the frequency by 3 s/time, we recorded the peak value until the mouse was pulled from the bar through the tail horizontally away.

### Biochemical analysis

The serum testosterone levels were detected by ELISA kits (Ruixing Biological, Quanzhou, China). We used the biochemical analyzer to detect glucose, cholesterol, high-density lipoprotein cholesterol, low-density lipoprotein cholesterol, and triglyceride in serum.

### Histological analysis

Eosin (H&E) staining and transmission electron microscopy were performed as previously described (Hou et al., 2020). Eosin (H&E) staining: tissues were incubated with fixed liquid (Servicebio, Wuhan, China) for at least 24 h. Then, tissues were embedded in paraffin, sectioned at 5 μm, and stained with hematoxylin–eosin. The CSA of myofibers was determined by ImageJ (NIH) software. Transmission electron microscopy: tissues were fixed in 2.5% glutaraldehyde solution (18 h) and fixed in osmium solution (2 h). Acetone gradient dehydration, acetone-resin (1:1) soaked (4 h), and embedding agent soaked (18 h). Then, tissues were placed in an embedding frame, embedded with resin, soaked, and polymerized at high temperatures for 48 h. Tissues were sectioned at 60 nm and stained with 2% uranyl acetate and lead citrate. Morphology of tissues was observed and photographed by the JEM-1400 microscope (JEOL, Tokyo, Japan; Liu et al., 2020).

### Quantitative polymerase chain reaction

According to previous studies (Hui et al., 2019; Zeng et al., 2019; Hui et al., 2020b), we used RNAiso Plus (Takara, Japan) to isolate total RNA from GA and testis. Then we reverse-transcribed RNA into cDNA using PrimeScript RT reagent Kit (Takara, Japan). We performed the qPCR using qTower 2.2 real-time PCR system (Analytik Jena, Germany). The primers were synthesized by Sangon Biotech (Shanghai, China) and listed in Supplementary Table 1.

### Sequencing of the gut microbiota

We collected the fecal samples using metabolic cages 24 h after the final training and saved them at −80°C until required. The 16S rRNA gene of the DNA sequence was analyzed by QILME2 software and the Illumina MiSeq platform (Illumina, San Diego, CA, United States; Bolyen et al., 2019). The α-diversity was analyzed via Ace, Chao, Sobs, Pd, Shannon, Shannoneven, Simpson, and Simpsoneven’s indices, and the Student’s *t*-test was used for statistical comparison. The principle coordinate analysis (PCoA) of β-diversity based on Hellinger was analyzed by analysis of similarities (ANOSIM). Then, we used the Linear Discriminant Analysis Effect Size (LEfSe) method to discover the bacterial biomarkers. We performed the LEfSe at a false discover rate (FDR) < 0.05 and linear discriminant analysis (LDA) score of >2.0.

### RNA sequencing analysis

We collected GA from C57BL/6J mice and extracted the total RNA using TRIzol^®^ Reagent. Then RNA quality was determined by 2100 Bioanalyser (Agilent Technologies, United States) and ND-2000 (NanoDrop Technologies, United States). The clean reads were obtained by FASTP software (Chen et al., 2018), and TPM indicated gene expression (Reads PerKilobases per Million reads). We selected the genes with at least 1.5-fold changes as differentially expressed.

### Statistical analysis

All experimental data were analyzed by GraphPad Prism 7 and SPSS 19.0 software. The data were presented as the mean ± SEM. The Student’s *t*-test was used to analyze data between 2 groups. One-way analysis of variance (ANOVA) was conducted for the comparison of more than 2 groups. Two-way ANOVA was used for multiple factors analysis (∗*p* < 0.05; ∗∗*p* < 0.01; ∗∗∗*p* < 0.001).

## Results

### High-intensity aerobic exercise improved skeletal muscle performance

**Figure 1:**
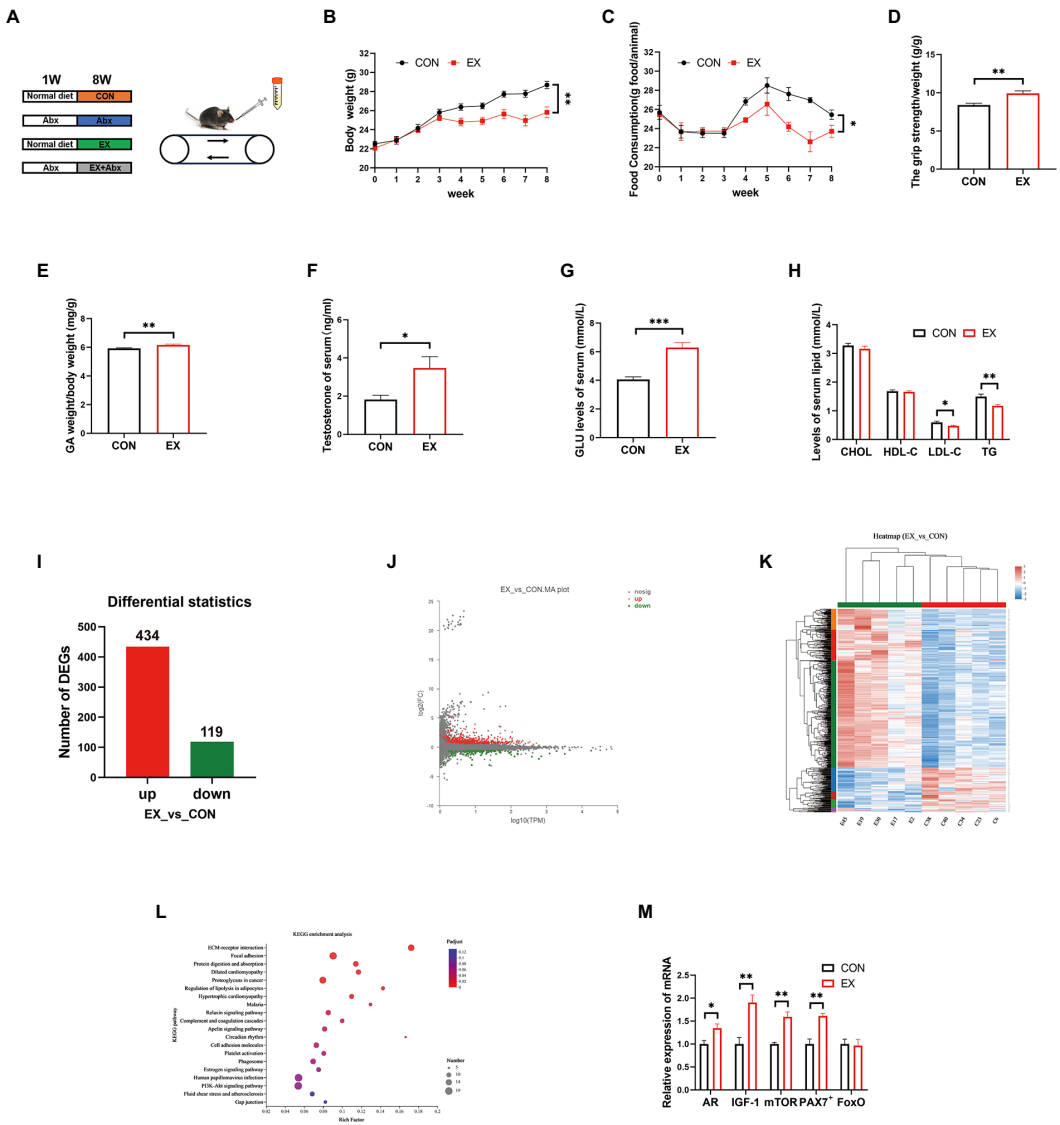
High loading intensity of aerobic exercise improved skeletal muscle performance (CON vs. EX). **(A)** Mice were subjected to treadmill training and antibiotic treatment for 8 weeks (CON, Abx, EX, and EX + Abx). **(B)** Body weight. **(C)** Food intake. **(D)** Grip strength. **(E)** GA percentage. **(F)** Serum testosterone levels. **(G)** Serum GLU levels. **(H)** Serum lipids levels. **(I)** Differentially expressed genes (DEGs). **(J)** MA plot. **(K)** Heat map of DEGs. **(L)** KEGG_pathway of DEGs. **(M)** The expression of related genes (AR, IGF-1, mTOR, PAX7+, and FoxO) by qPCR (∗*p* < 0.05; ∗∗*p* < 0.01; ∗∗∗*p* < 0.001).

To explore the effect of exercise on C57BL/6J mice, mice were subjected to treadmill running for 8 weeks (Figure 1A). The EX group had significantly less body weight (Figure 1B) and food intake (Figure 1C) than the CON group. However, exercise significantly improved the grip strength (Figure 1D) and GA percentage (Figure 1E) of mice in the EX group, which was positively correlated with testosterone levels in serum (Figure 1F) and cecum content (Supplementary Figure 1A). The testis percentage (Supplementary Figure 1B) and serum lactate levels (Supplementary Figure 1C) were the same between CON and EX groups. Besides, exercise could significantly increase serum GLU levels (Figure 1G) and low serum lipids levels (Figure 1H), indicating that glycogen reserve was elevated in the EX group. Moreover, there were no significant differences in serum alanine aminotransferase (ALT) and aspartate aminotransferase (AST) levels between the 2 groups (CON and EX; Supplementary Figures 1D,E). These results indicated that high-intensity aerobic exercise had beneficial effects on C57BL/6J mice.

To further confirm the effect of exercise on gene expression in GA, we performed RNA sequencing. The RNA quality was shown in Supplementary Figure 2A and Supplementary Table 2. There are significant differences in gene expression between CON and EX groups (Supplementary Table 3; Figure 1I; and Supplementary Figure 2B). The MA plot was performed by R package ggplot2 (Figure 1J) and the heatmap was generated by DESeq2 (Figure 1K). The KEGG analysis was further performed on the differential genes, revealing an improvement in the lipolysis signaling pathway (Figure 1L; Supplementary Table 4) under exercise. Moreover, exercise improved the expression of skeletal muscle hypertrophy-related genes (Figure 1M). Altogether, these data confirmed that high-intensity aerobic exercise could alter the expression of related genes in GA.

**Figure 2:**
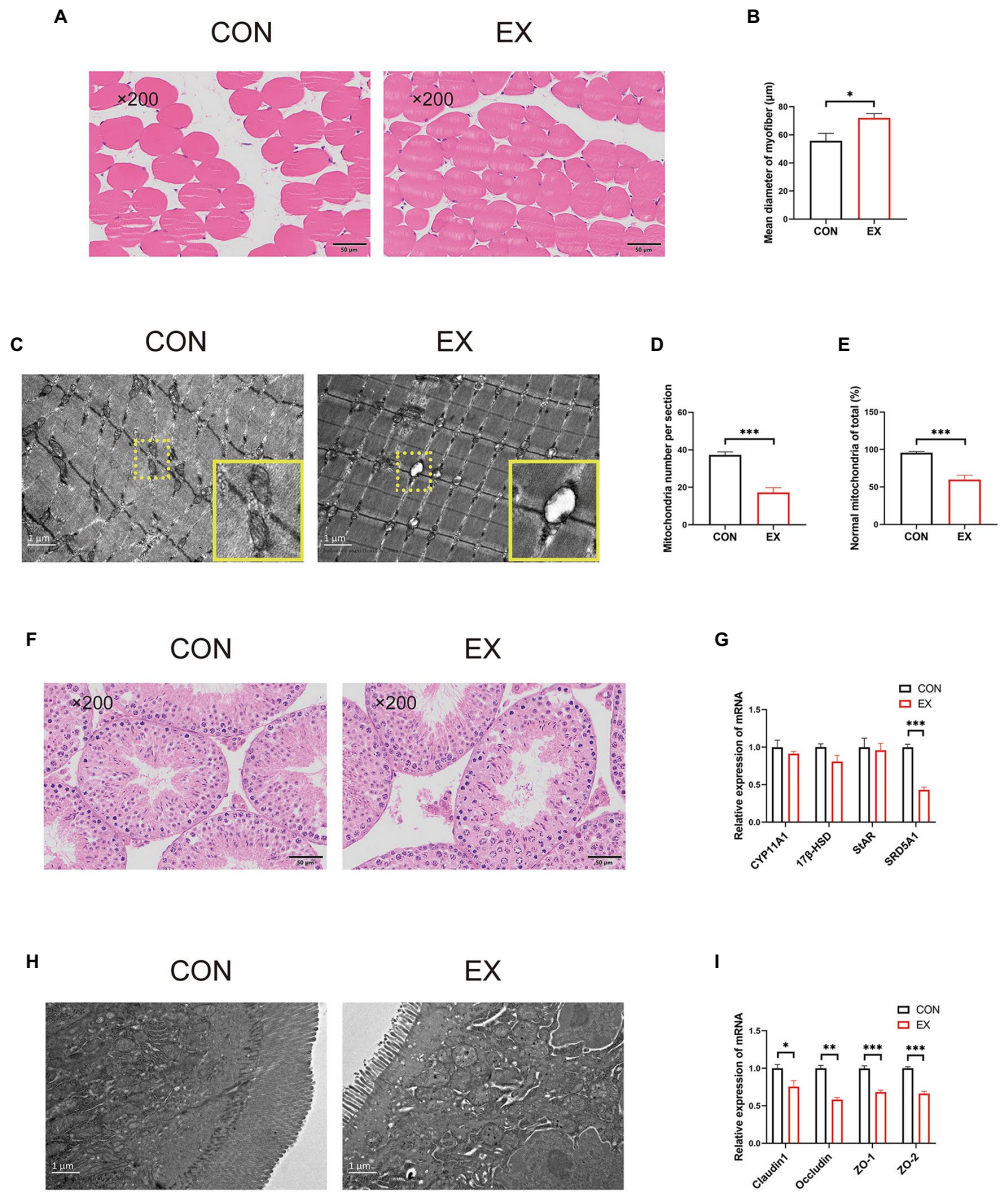
High-intensity aerobic exercise improved the CSA of the skeletal muscle (CON vs. EX). **(A)** HE staining of the skeletal muscle. **(B)** Mean diameter of the myofiber. **(C–E)** Mitochondria oxidative stress damage to skeletal muscle through transmission electron microscopy. **(F)** HE staining of the testis. **(G)** The expression of StAR, CYP11A,17β-HSD, and SRD5A1 by qPCR. **(H)** Transmission electron microscopy of the small intestine. **(I)** The expression of Claudin1, Occludin, ZO-1, and ZO-2 by qPCR (∗*p* < 0.05; ∗∗*p* < 0.01; ∗∗∗*p* < 0.001).

The CSA of the skeletal muscle had been increased in the EX group (Figures 2A,B). Unexpectedly, we found mitochondria oxidative stress damage of skeletal muscle in the EX group through transmission electron microscopy (Figures 2C,E). Besides, there was no obvious difference in the testis between the two groups by HE staining (Figure 2F). Exercise could not alter the expression of StAR, CYP11A1, and 17β-HSD (Figure 2G), indicating that the production of testosterone was not changed. Unexpectedly, the expression of barrier-related genes was substantially decreased in the EX group (Supplementary Figure 2C). Moreover, through transmission electron microscopy, exercise could substantially cause damage to the intestinal in the EX group (Figure 2H) and substantially decreased the expression of barrier-related genes (Figure 2I). These results indicated that high-intensity aerobic exercise could induce skeletal muscle hypertrophy, but also caused some damage to the body during the exercise injury period.

### High-intensity aerobic exercise remodeled the gut microbiota’s structure

**Figure 3:**
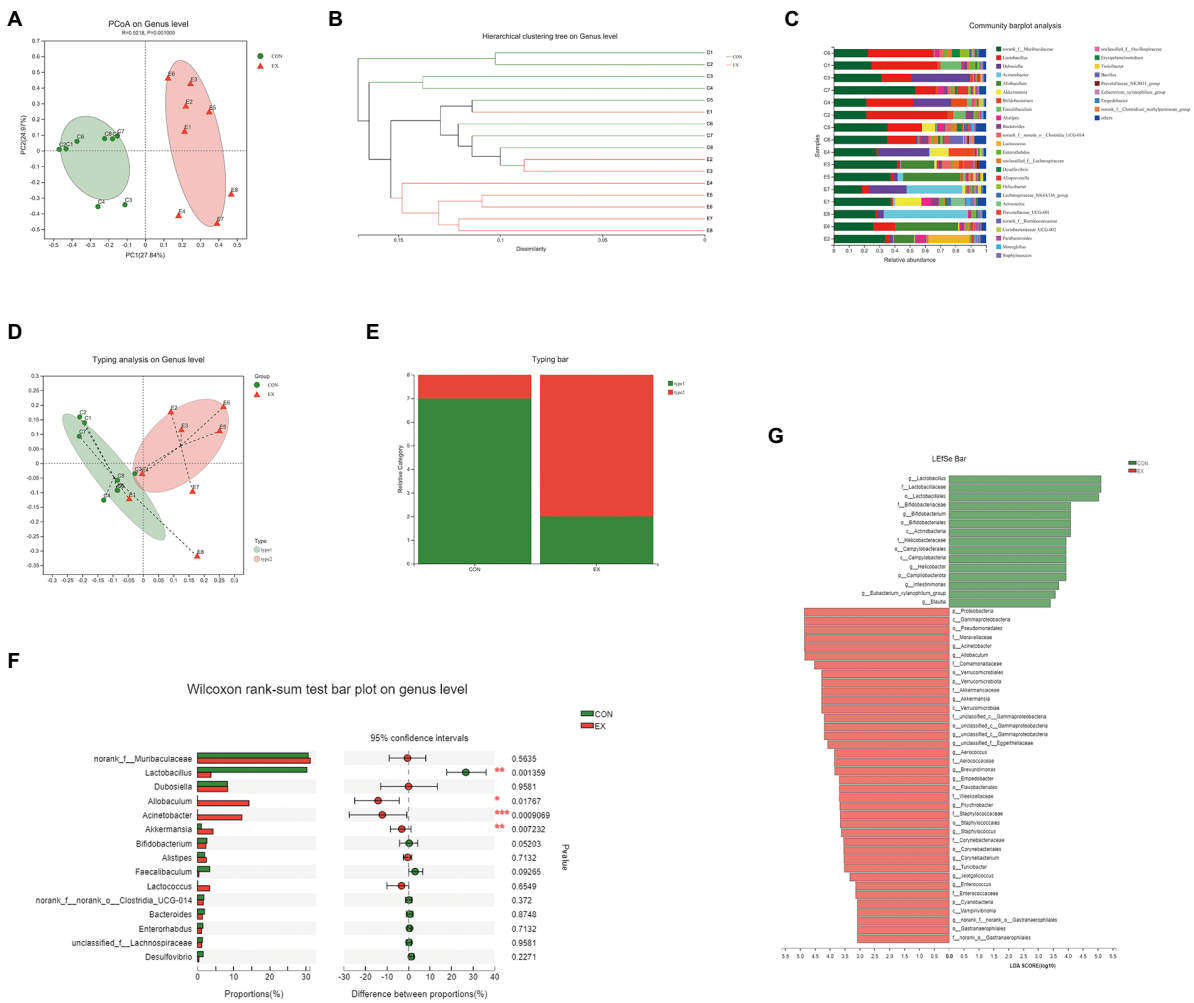
High-intensity aerobic exercise remodeled the gut microbiota structure (CON vs. EX). **(A)** A clear separation was observed by principal coordinate analysis (PCoA) based on the hellinger. **(B)** Hierarchical cluster analysis based on unweighted_unifrac. **(C)** Species composition of 16 samples on the genus level. **(D,E)** 16 samples were clustered into 2 distinct enterotypes using abund_jaccard. **(F)** The Wilcoxon rank-sum test bar plot on the genus level. **(G)** Results of the LEfSe analysis.

To explore the effects of exercise on the gut microbiota’s structure, we collected the fecal samples from CON and EX groups after the final training and performed 16S rRNA profiling. We used the Illumina platform (Majorbio, China) to generate 1,346,823 sequences ranging from 68,897 to 114,648 per sample (*n* = 16 in total) representing 10,114 ASVs (Supplementary Table 5). There were no significant differences in gene enrichment, species abundance, and community evenness on the genus level by Pan analysis (Supplementary Figure 3A) and Rank abundance curve (Supplementary Figure 3B). A comparison of alpha diversity based on the genus level were no statistical difference (Supplementary Figures 3C–J; Supplementary Table 6). Moreover, we found a clear separation based on the hellinger by principal coordinate analysis (PCoA; Figure 3A). The hierarchical cluster analysis was performed based on unweighted_unifrac (Figure 3B), suggesting that exercise altered the gut microbiota’s structure.

On the genus level, the species composition of samples was shown in Figure 3C. Then, all samples were clustered into 2 distinct enterotypes at the genus level using abund_jaccard (Figures 3D,E). The major contributors in type 1 (green) were *Muribaculaceae* (30.31%) and *Lactobacillus* (26.18%; Supplementary Table 7). And the key contributors in type 2 (red) were *Muribaculaceae* (30.11%) and *Allobaculum* (15.61%; Supplementary Table 8). The percentage of type 1 was 87.5% in the CON groups, and type 2 (75%) was more prevalent in the EX group. Moreover, exercise increased the abundance of *Allobaculum*, *Acinetobacter*, and *Akkermansia*, whereas reduced *Lactobacillus* (Figure 3F). The LEfSe analysis results indicated that exercise caused an increase in the relative abundance of members of the family *Akkermansiaceae*, genus *Akkermansia*, and genus *Allobaculum* (Figure 3G). Taken together, these results indicated that high-intensity aerobic exercise could modify the gut microbiota’s structure.

### The beneficial effect of high-intensity aerobic exercise disappeared after the elimination of the gut microbiota

**Figure 4:**
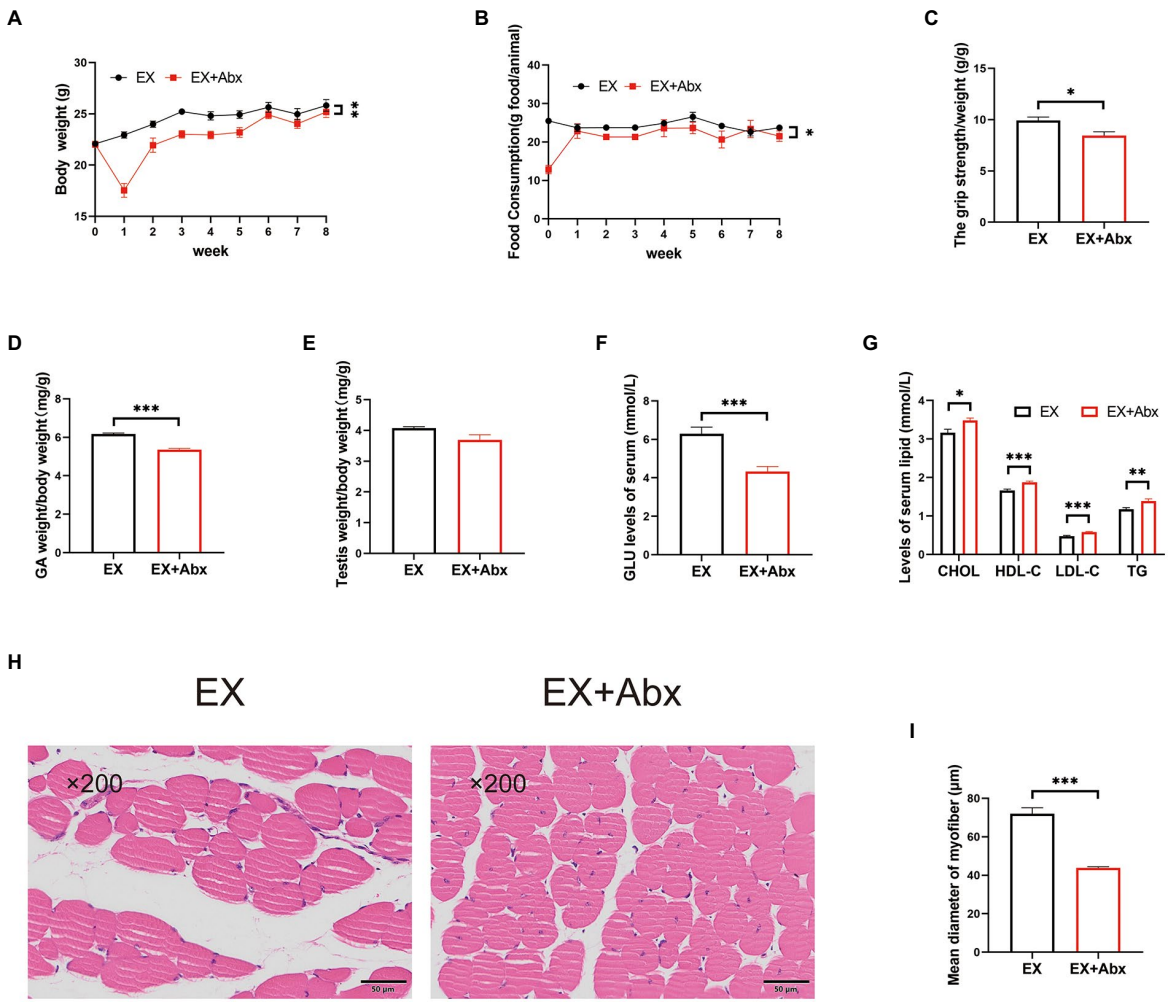
The high-intensity aerobic exercise disappeared after the elimination of the gut microbiota (EX vs. EX+Abx). **(A)** Body weight. **(B)** Food intake. **(C)** Grip strength. **(D)** GA percentage. **(E)** Testis percentage. **(F)** Serum GLU levels. **(G)** Serum lipids levels. **(H)** Histological staining of the skeletal muscle. **(I)** Mean diameter of the myofiber (∗*p* < 0.05; ∗∗*p* < 0.01; ∗∗∗*p* < 0.001).

To observe the key role of the gut microbiota, we performed the Abx treatment (Figure 1A). The stool DNA concentration from stool samples was significantly decrease after Abx treatment (Supplementary Figure 4A). Compared with the normal groups, the Abx groups had significantly less body weight (Figure 4A; Supplementary Figure 4B) and food intake (Figure 4B; Supplementary Figure 4C). Besides, the grip strength (Figure 4C) and GA percentage (Figure 4D; Supplementary Figure 4D) of mice were significantly lower in the Abx groups. There was no obvious difference in testis percentage between EX and EX+Abx groups (Figure 4E). However, the serum testosterone levels was significantly lower in EX+Abx group compared with the EX group (Supplementary Figure 4E). Moreover, the Abx treatment could significantly decrease serum GLU levels (Figure 4F; Supplementary Figure 4F) and improve serum lipids levels (Figure 4G; Supplementary Figure 4G), showing that gut microbiota played a crucial role in glucose and lipid metabolism. Compared with the EX group, the Abx treatment decreased the CSA of the skeletal muscle by HE staining (Figures 4H,I). These results indicated that the beneficial effect of exercise disappeared after the elimination of the gut microbiota.

### Gut microbiota played a key role in skeletal muscle performance

**Figure 5:**
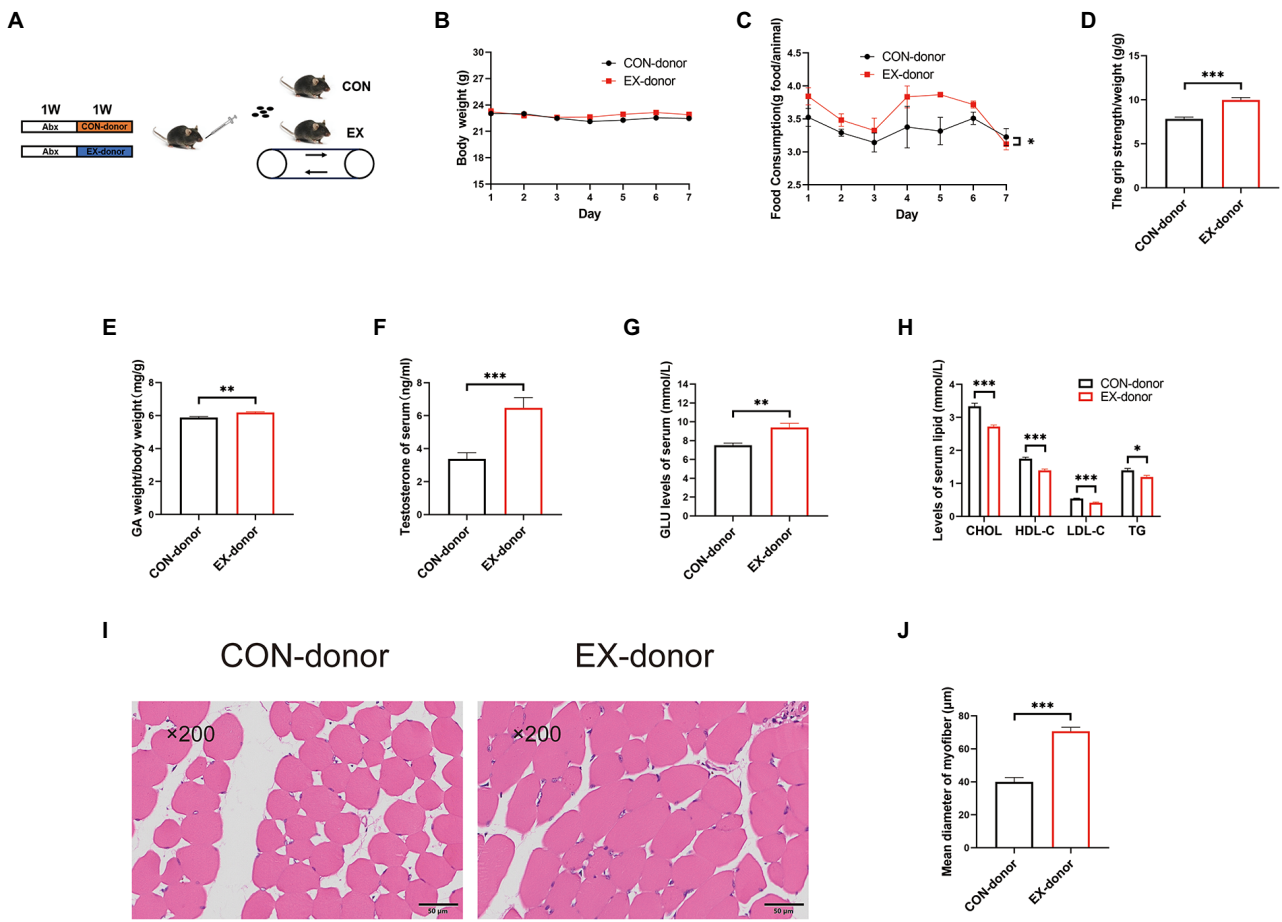
The gut microbiota played a key role in skeletal muscle performance (CON-donor, EX-donor). **(A)** Mice were subjected to FMT for 1 week. **(B)** Body weight. **(C)** Food intake. **(D)** Grip strength. **(E)** GA percentage. **(F)** Testis percentage. **(G)** Serum GLU levels. **(H)** Serum lipids levels. **(I)** HE staining of the skeletal muscle. **(J)** Mean diameter of the myofiber (∗*p* < 0.05; ∗∗*p* < 0.01; ∗∗∗*p* < 0.001).

To further prove the critical role of gut microbiota, we performed the FMT experiment (Figure 5A). After 1 week FMT experiment, the stool DNA concentration from stool samples was restored (Supplementary Figure 4A). The body weight was the same between the 2 groups (Figure 5B). but the food intake was increased in the EX-donor group (Figure 5C). Moreover, compared with the CON-donor group, the grip strength (Figure 5D) and GA percentage (Figure 5E) were improved in the EX-donor group. The serum testosterone and GLU levels were higher (Figures 5F,G), but the serum lipids levels were lower in the EX-donor group (Figure 5H), indicating that exercise could lower blood lipids. Through HE staining microscopy, the CSA of the skeletal muscle was increased in the EX-donor group (Figures 5I,J). These results suggested that gut microbiota played a key role in skeletal muscle performance.

## Discussion

The quest to increase skeletal muscle performance is widely pursued by athletes. Exercise is widely used to increase skeletal muscle performance, especially for athletes (Camera et al., 2016; Schoenfeld et al., 2016; McKendry et al., 2021). Resistance exercise could significantly induce muscle hypertrophy. It has been reported that the CSA of muscle fiber has increased by more than 50% under resistance exercise (Hubal et al., 2005; Bamman et al., 2007). However, the beneficial effect of high-intensity aerobic exercise on skeletal muscle remains to be elucidated. In our study, we found that the 8-week high-intensity aerobic exercise could improve muscle performance by increasing the CSA of GA.

Testosterone is a key regulator of protein metabolism in muscle (Tipton and Wolfe, 2001). Accumulating evidence indicates that testosterone participates in satellite cell number, muscle mass, and grip strength (Serra et al., 2013). Long-term testosterone replacement therapy (TRT) improves muscle mass in humans with low serum testosterone levels (Kruse et al., 2020). Our results showed that high-intensity aerobic exercise could improve the serum testosterone levels, but not alter the testosterone production in the testis. These results indicated a new endogenous pathway to produce testosterone except the hypothalamic–pituitary-testicular axis (HPA).

It has been repeatedly shown that gut microbiota is related to skeletal muscle performance (Lahiri et al., 2019; Mailing et al., 2019; Scheiman et al., 2019). The germ-free mice show atrophy of skeletal muscle, which could be reversed by FMT (Hsu et al., 2015; Huang et al., 2019). Gut microbiota is the primary regulator of testosterone metabolism, which could convert T-G to T (Colldén et al., 2019). *Akkermansia*, considered to be a promising candidate for probiotics, might be related to testosterone metabolism (Zhang et al., 2021). Importantly, testosterone produced by the gut microbiota could improve muscle performance by enhancing skeletal muscle protein synthesis (Rossetti et al., 2017). At the genus level, we further demonstrated that exposure to the long-term high loading intensity of aerobic exercise increased the abundance of *Allobaculum* and *Akkermansia*, accompanied by testosterone production in C57BL/6 mice.

**Figure 6:**
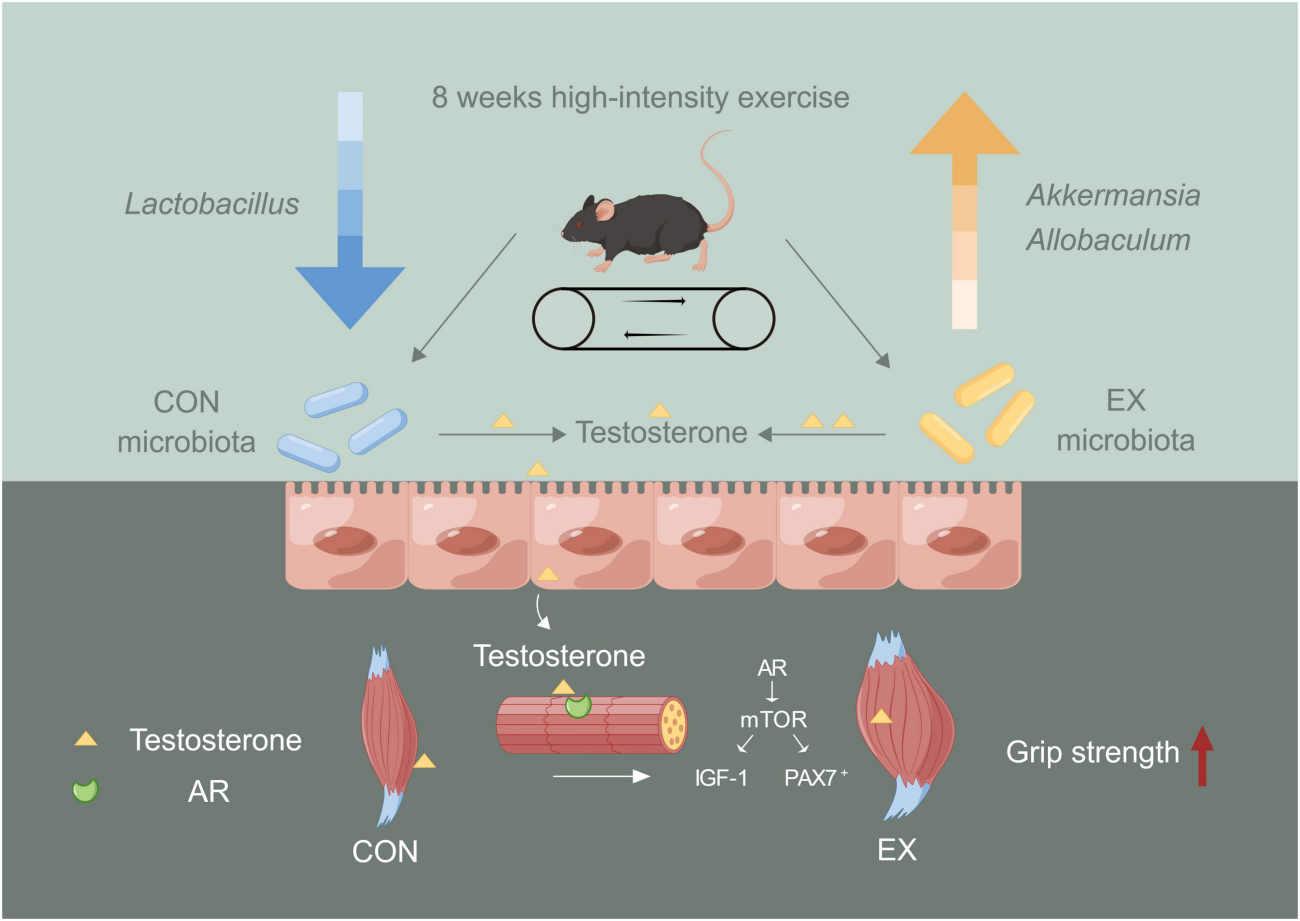
The role of gut microbiota on skeletal muscle performance by high-intensity aerobic exercise (by Figdraw). The long-term (8 weeks) high loading intensity of aerobic exercise improves serum testosterone levels and skeletal muscle performance by remodeling the gut microbiota’s structure (Lactobacillus, Allobaculum, and Akkermansia).

Our results revealed that the high-intensity aerobic exercise remodeled the gut microbiota’s structure and improved skeletal muscle performance via the gut microbiota-testosterone axis (Figure 6). We provided new insights into improving serum testosterone levels through endogenous pathways. However, exercise might cause mitochondrial oxidative stress injury in the skeletal muscle during the exercise injury period, further investigation was needed to prevent injury induced by the high-intensity exercise. Exercise combined with sports nutrition supplements might be a better choice for athletes. Further study should be required to observe the beneficial effect of high-intensity aerobic exercise, sports nutrition supplements, and gut microbiota on athletes.

## Conclusion

Overall, our study revealed that long-term high loading intensity of aerobic exercise appeared to remodel the gut microbiota’s structure (*Lactobacillus, Allobaculum*, and *Akkermansia*), affected testosterone metabolism, and improved skeletal muscle performance. And we provided new insights into the role of the gut microbiome in testosterone metabolism.

## Data availability statement

The data presented in the study are deposited in the NCBI repository, accession number PRJNA 871043 and PRJNA872497.

## Ethics statement

The animal study was reviewed and approved by the Animal Care and Use Committee of the Army Medical University.

## Author contributions

LZ designed the study, instructed all experiments, and drafted the manuscript. HL carried out the data analysis. LR, GT, and HS assisted in running training and performing the experiments. MM, LY, JZ, and QZ provided many suggestions on the articles and obtained funding. All authors contributed to the article and approved the final manuscript.

## Funding

This work was supported by the grants from the Key Projects for Scientific Research (AWS17J014 and HZ2022-158).

## Conflict of interest

The authors declare that the research was conducted in the absence of any commercial or financial relationships that could be construed as a potential conflict of interest.

## Publisher’s note

All claims expressed in this article are solely those of the authors and do not necessarily represent those of their affiliated organizations, or those of the publisher, the editors and the reviewers. Any product that may be evaluated in this article, or claim that may be made by its manufacturer, is not guaranteed or endorsed by the publisher.

## Abbreviations

Abx: antibiotic cocktail
HPA: hypothalamic-pituitary-testicular axis
DHT: dihydrotestosterone
T-G: glucuronidated testosterone
GA: gastrocnemius muscles
FMT: fecal microbiota transplantation
CSA: cross-sectional area
ANOVA: one-way analysis of variance
ALT: alanine aminotransferase
AST: aspartate aminotransferase
PCoA: principal coordinate analysis
DEGs: differentially expressed genes
TRT: testosterone replacement therapy
