# Supplementary figures and images for: Long-term high loading intensity of aerobic exercise improves skeletal muscle performance via the gut microbiota-testosterone axis
Source: Front Microbiol. 2022 Dec 21;13:1049469. doi: 10.3389/fmicb.2022.1049469 (PMC9811821; doi:10.3389/fmicb.2022.1049469)

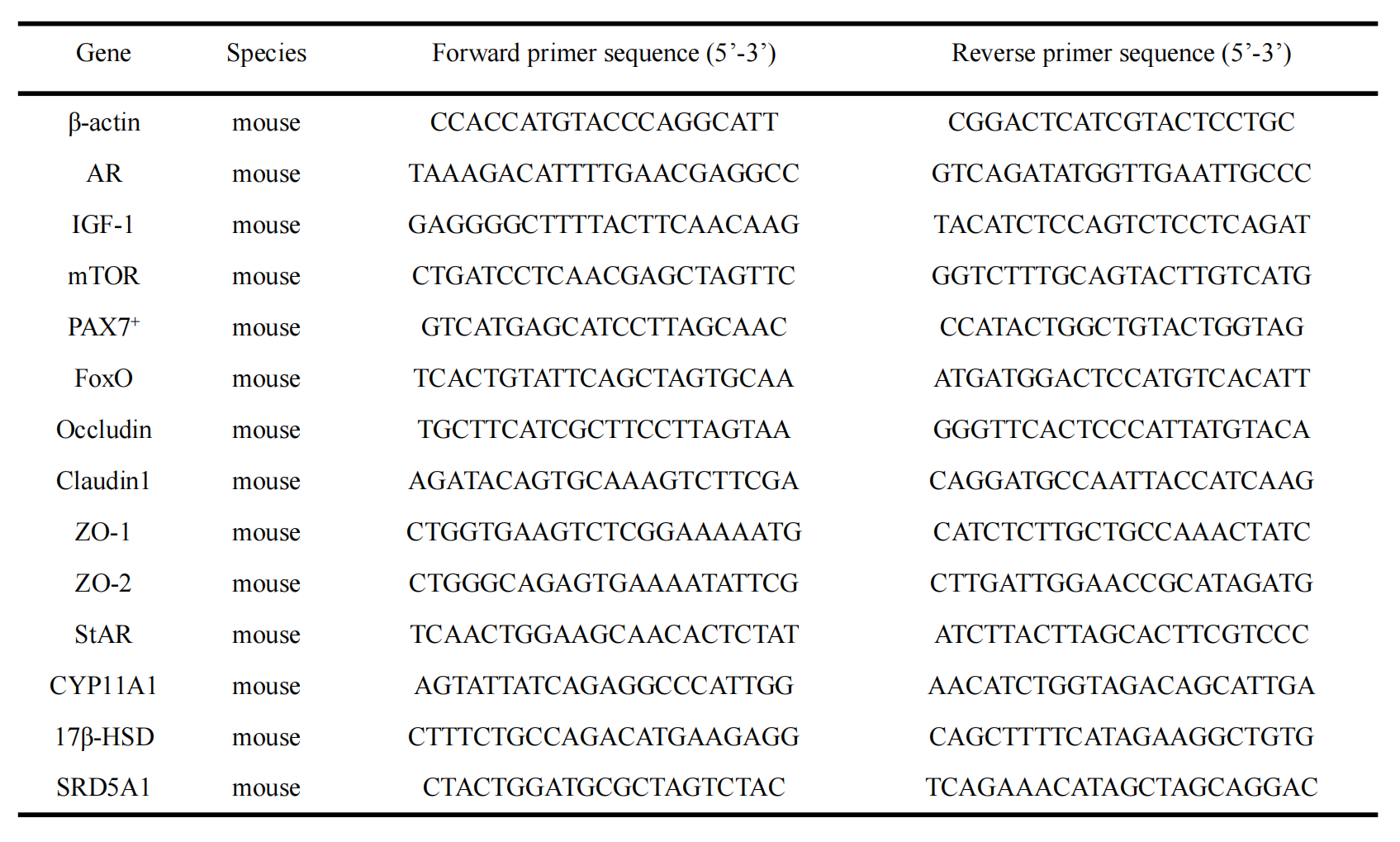

Supplement: Supplementary file 1 [file Data_Sheet_1.ZIP › Table/Table S1.png]
