# Supplementary material for: Long-term high loading intensity of aerobic exercise improves skeletal muscle performance via the gut microbiota-testosterone axis
Source: Front Microbiol. 2022 Dec 21;13:1049469. doi: 10.3389/fmicb.2022.1049469 (PMC9811821; doi:10.3389/fmicb.2022.1049469)
Supplement: Supplementary file 2 [file Data_Sheet_2.docx]

***Supplementary Material***

**1.1** **Supplementary Tables**

**
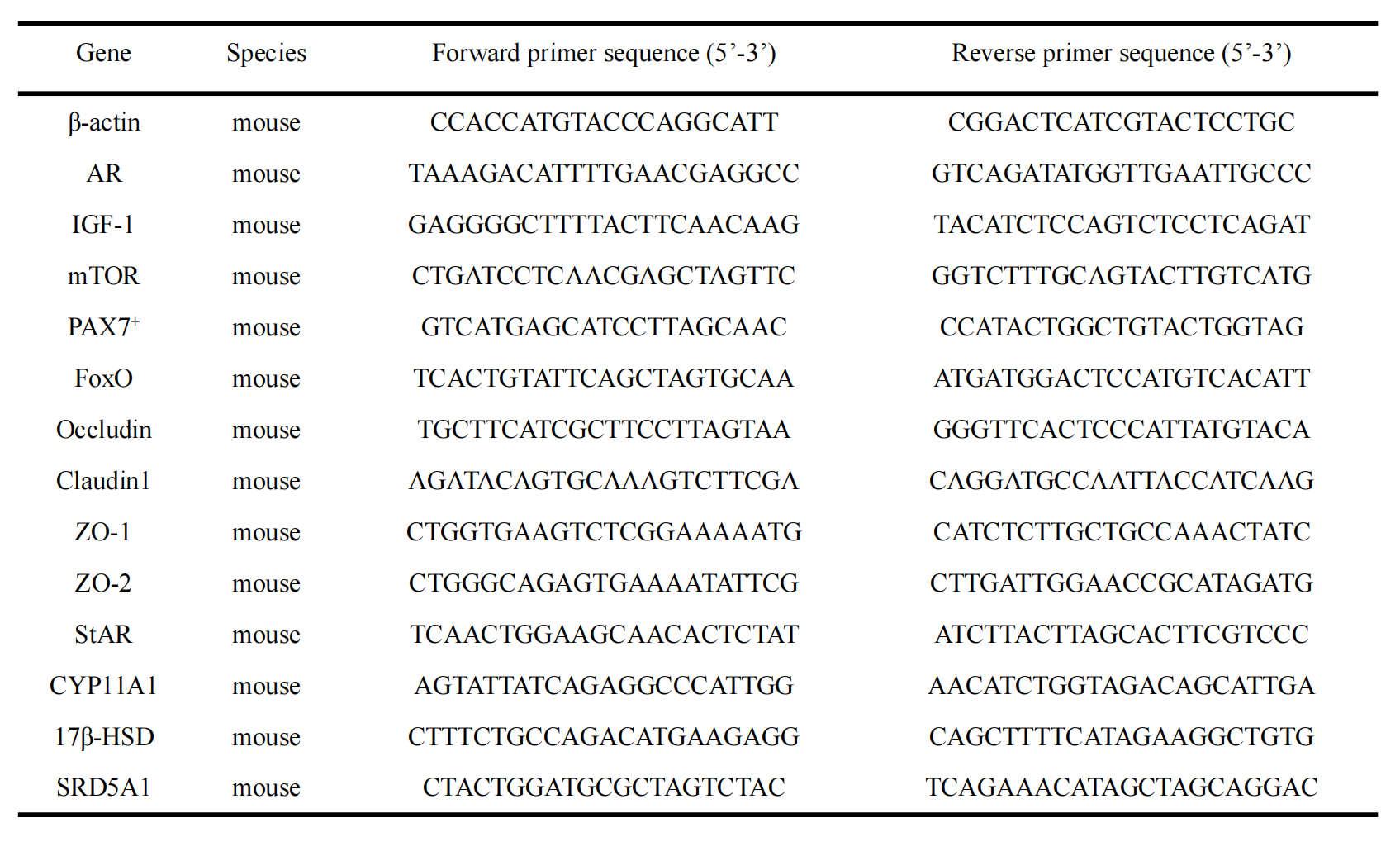
**

**Table 1. Primer sequences of target genes for qPCR.**

**1.2 Supplementary Figures**

**1.2.1 Supplementary Figure 1**

**
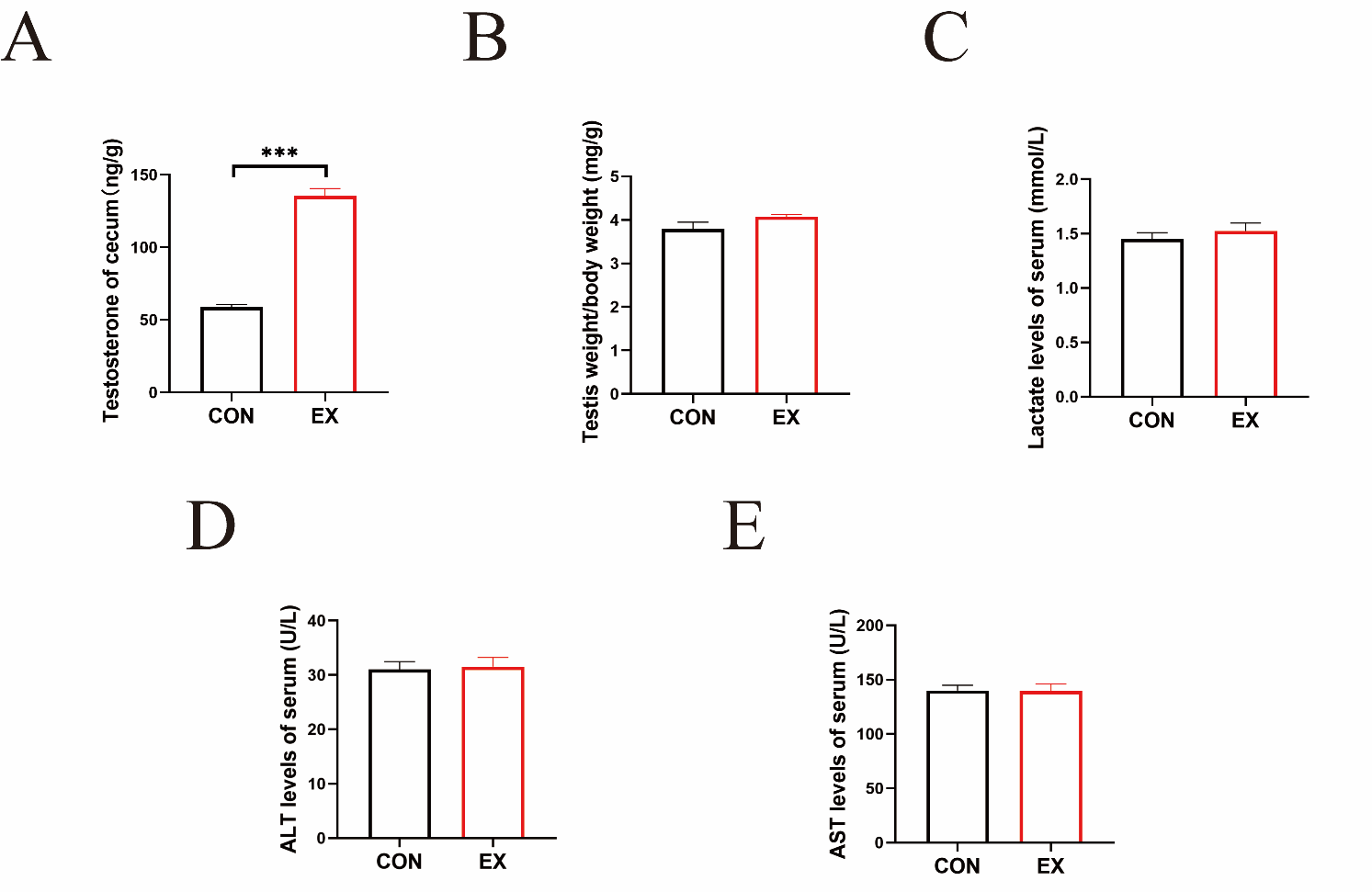
**

**Supplementary Figure 1.** The effect of high-intensity aerobic exercise on C57BL/6J mice (CON vs EX). (A) Cecum testosterone levels. (B) Testis percentage. (C) Serum lactate levels. (D) Serum ALT levels. (E) Serum AST levels (∗p < 0.05; ∗∗p < 0.01; ∗∗∗p < 0.001).

**1.2.2 Supplementary Figure 2**

**
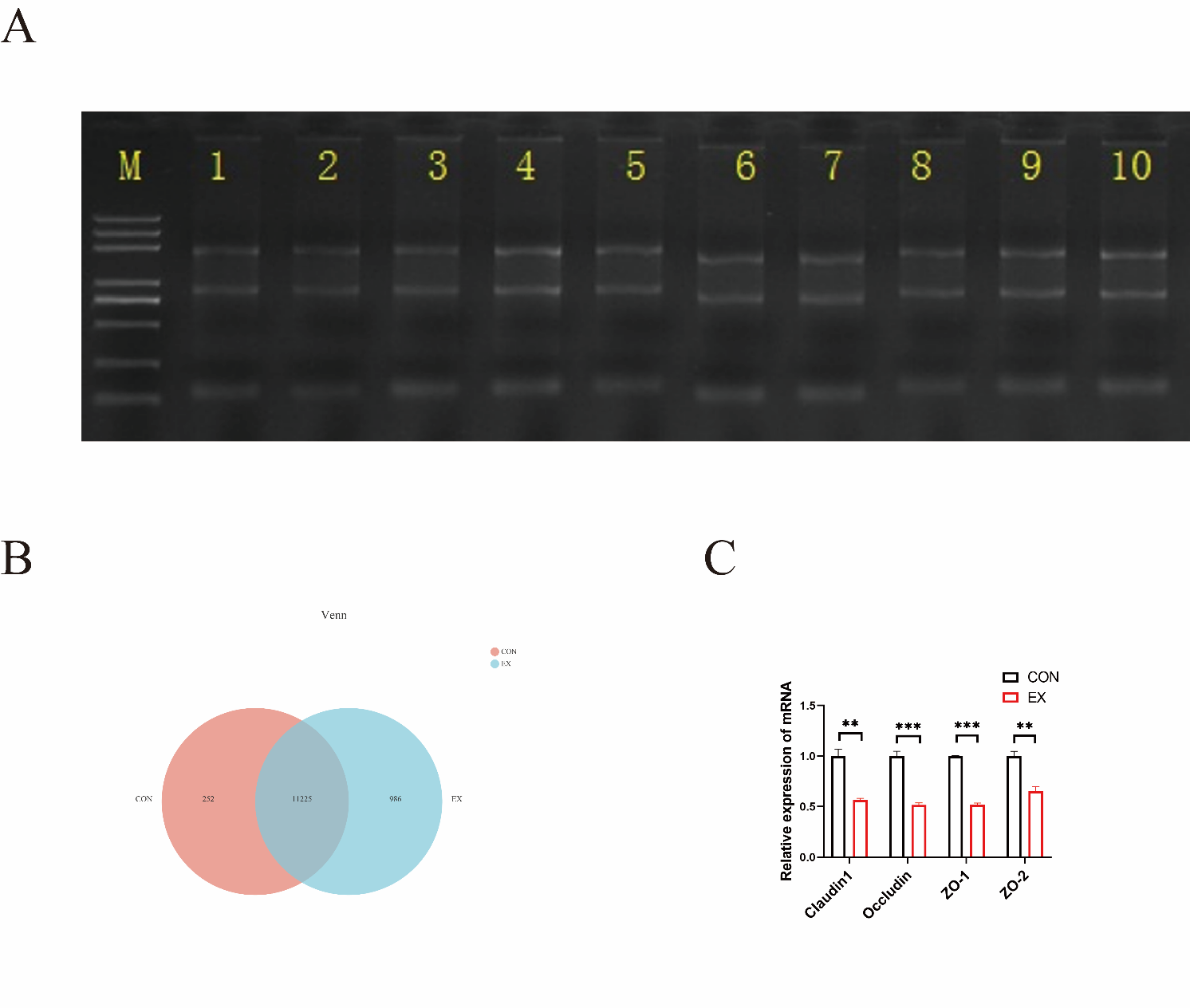
**

**Supplementary Figure 2.** The gene expression in CON and EX groups (CON vs EX). (A) RNA quality of skeletal muscle. (B) Venn plot. (C) The expression of related gene (Claudin1, Occludin, ZO-1, and ZO-2) by qPCR (∗p < 0.05; ∗∗p < 0.01; ∗∗∗p < 0.001).

**1.2.3 Supplementary Figure 3**

**
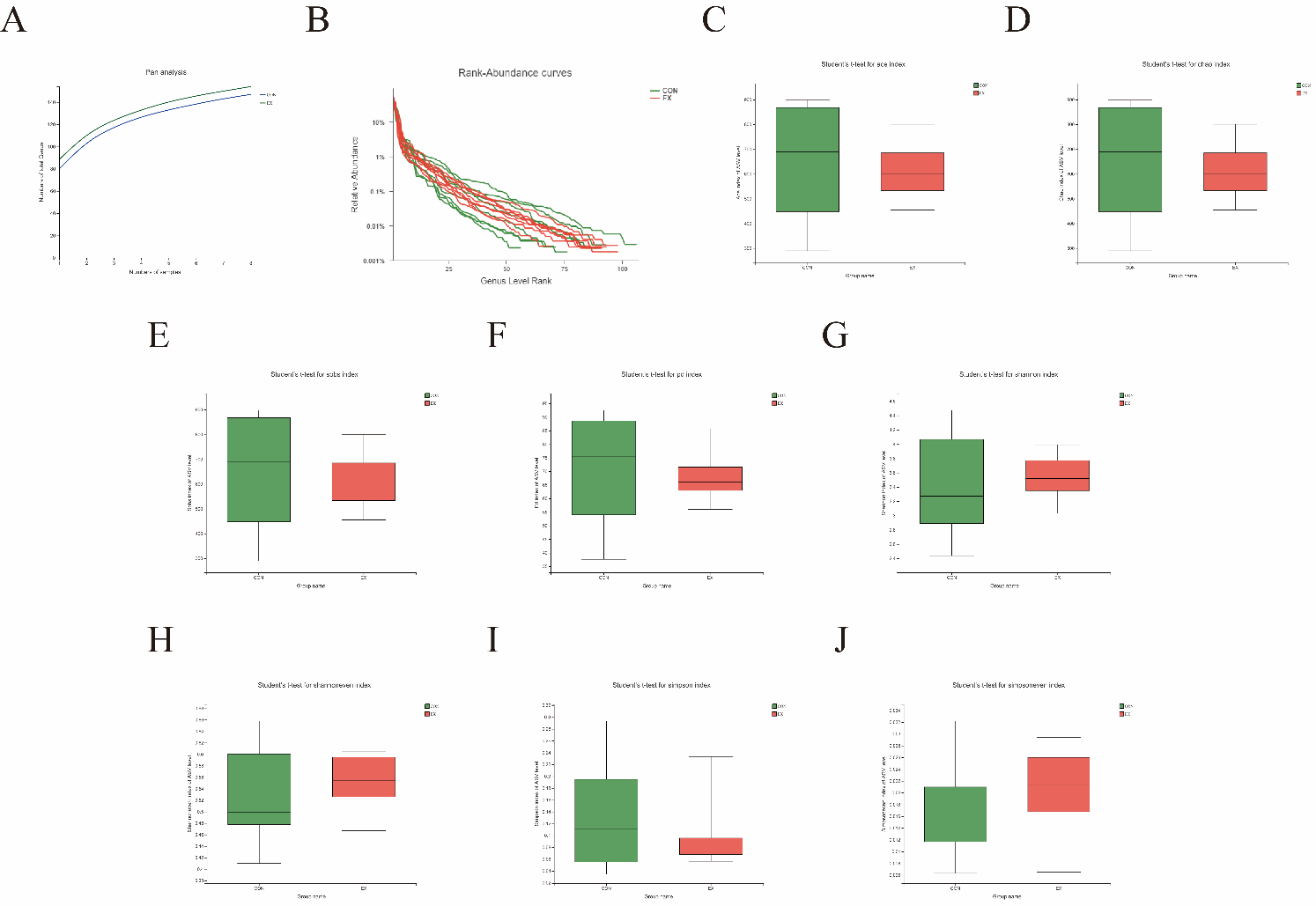
**

**Supplementary Figure 3.** High-intensity aerobic exercise remodeled the structure of gut microbiota (CON vs EX). (A) Gene enrichment by Pan analysis on the genus level. (B) The species abundance and community evenness by the Rank abundance curve on the genus level. (C-J) A comparison of alpha diversity ( Ace, Chao, Sobs, Pd, Shannon, Shannoneven, Simpson, and Simpsoneven) (∗p < 0.05; ∗∗p < 0.01; ∗∗∗p < 0.001).

**1.2.4 Supplementary Figure 4**

**
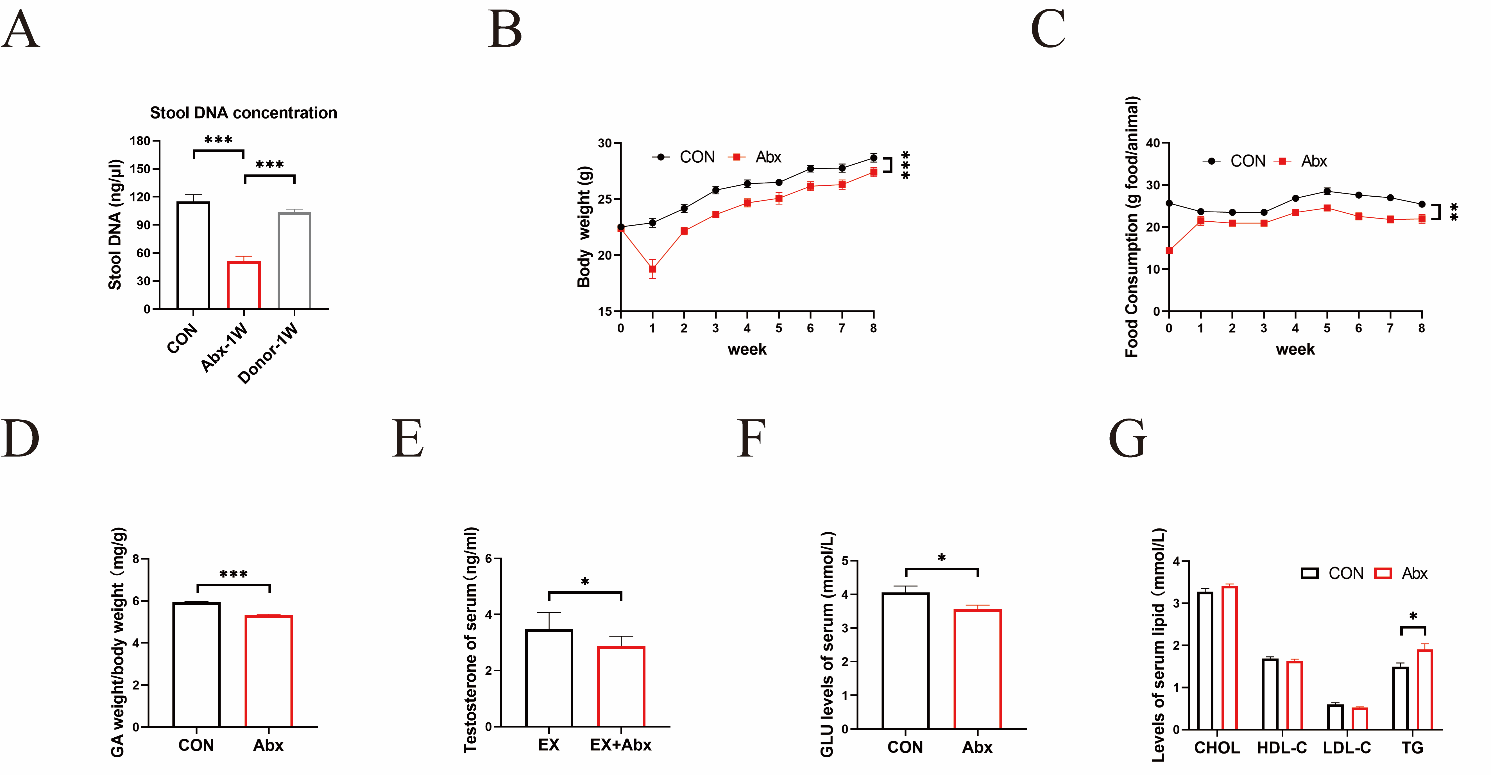
**

**Supplementary Figure 4.** The Abx treatment decreased skeletal muscle performance (CON vs Abx). (A) Stool DNA concentration. (B) Body weight. (C) Food intake. (D) GA percentage. (E) Serum testosterone levels. (F) Serum GLU levels. (G) Serum lipids levels (∗p < 0.05; ∗∗p < 0.01; ∗∗∗p < 0.001).
